# Diphenhydramine inhibits voltage-gated proton channels (Hv1) and induces acidification in leukemic Jurkat T cells- New insights into the pro-apoptotic effects of antihistaminic drugs

**DOI:** 10.1080/19336950.2017.1331799

**Published:** 2017-05-17

**Authors:** Agustín Asuaje, Pedro Martín, Nicolás Enrique, Leandro Agustín Díaz Zegarra, Paola Smaldini, Guillermo Docena, Verónica Milesi

**Affiliations:** Instituto de Estudios Inmunológicos y Fisiopatológicos (IIFP, CONICET—Universidad Nacional de la Plata), Fac. de Ciencias Exactas, Universidad Nacional de La Plata, La Plata, Argentina

**Keywords:** antihistaminic, apoptosis, cancer, diphenhydramine, HVCN1, intracellular pH, leukemia, proton channels

## Abstract

An established characteristic of neoplastic cells is their metabolic reprogramming, known as the Warburg effect, with greater reliance on energetically less efficient pathways (such as glycolysis and pentose phosphate shunt) compared with oxidative phosphorylation. This results in an overproduction of acidic species that must be extruded to maintain intracellular homeostasis. We recently described that blocking the proton currents in leukemic cells mediated by Hv1 ion channels triggers a marked intracellular acidification and apoptosis induction. Moreover, histamine H1-receptor antagonists were found to induce apoptosis in tumoral cells but the mechanism is still unclear. By using Jurkat T cells, we now show how diphenhydramine inhibits Hv1 mediated currents, inducing a drop in intracellular pH and cellular viability. This provides evidence of a new target structure responsible of the known pro-apoptotic action of antihistaminic drugs.

## Introduction

Diphenhydramine (DPH) is a well-known first-generation histamine H1-receptor antagonist, commonly used in treatment of allergic diseases. Its usefulness is principally related to a decrease of histamine effects produced during the hypersensitivity reaction. Furthermore, it has been reported that DPH has other molecular targets such as muscarinic and NMDA glutamatergic receptors which explain most of its adverse reactions.^1,2^

On the other hand, Jangi *et al.* clearly described that DPH promoted leukemic Jurkat T cell death by apoptosis at higher doses than those needed for its antihistaminic action.^3^ The authors later found similar results in other malignant cell lines.^4,5^ Although these observations are reproduced with several antihistaminic drugs, other evidence contradicts the involvement of the H1 receptor pathway in the apoptosis mechanism as it is exerted even in presence of exogenous histamine, α-fluoromethylhistidine treatment or H1 receptor knockdown. Moreover, antihistaminic pro-apoptotic action affected neither cAMP nor cGMP intracellular levels, both widespread second messengers.^4,5^

In addition, it is widely known that neoplastic cells show significant changes in glucose metabolism relying on glycolytic and pentose phosphate pathways instead of oxidative phosphorylation for energy production.^6,7^ This fact leads to acidic species overproduction and therefore a strong need for proton extrusion to avoid intracellular acidification.^8^ In this sense, several proton transporters were found overexpressed in tumor cells and they have been a matter of study in recent years. MCT, NHE, CA, NBC and V-ATPase (see abbreviations below) are the structures that most captured the attention in this field.^9,10^ Although less studied, the Hv1 channel has a strong capacity of restoring intracellular pH after heavy acid loads; and as it is a passive transport pathway, its activity does not require any expense of energy.^11^ This channel has been found upregulated in several tumor cell lines^12-16^ and clinical isolates, correlating with poor prognosis in human breast and colorectal cancer.^17,18^ In the same manner, other ion channels are emerging as key players in tumoral development.^19,20^

Recently our group showed that Hv1 channel in Jurkat T cells is indispensable for pHi regulation as its inhibition exerted by Zn^2+^ or ClGBI, both proved Hv1 blockers,^21^ induced an immediate drop in basal pHi along with significant impairment of the cell's capacity for restoring intracellular pH after heavy acid loads. Moreover, we observed that upon Hv1 channel blockade, acidification progressed from a pH around 7.2 to values below 6.8 and promoting cell death by apoptosis.^22^

In addition, Kim et al. have recently reported that DPH and chlorpheniramine induce a significant inhibitory effect on Hv1 currents in murine BV12 microglial cells.^23^ These authors also assert that Hv1 inhibition is independent of histamine receptor activity, as histamine does not modify any property of Hv1 currents.

Overall, we hypothesized that, if DPH is able to inhibit Hv1 in human Jurkat T cells (likewise it does in microglial cells), it could induce cell acidification as an early event of its known pro-apoptotic effect.^3^ In this work we present electrophysiological data showing the inhibition by DPH on Hv1 currents in Jurkat T cells and its action on intracellular pH.

## Results

**Figure 1.**
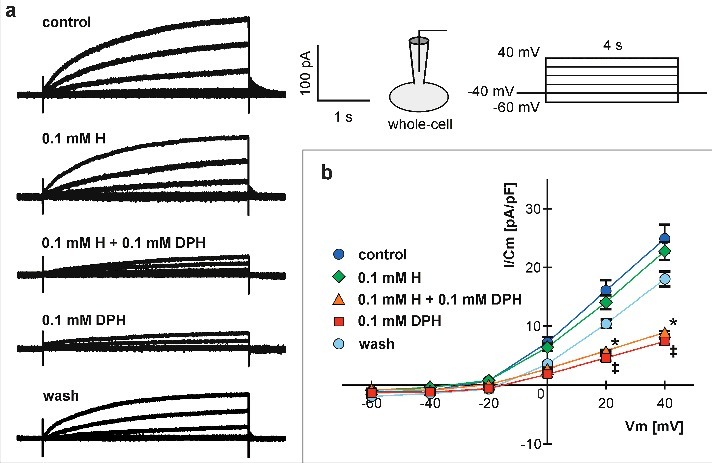
DPH inhibitory effects on Hv1 whole-cell currents. (a) Superimposed typical whole-cell currents recorded in response to 4-s long pulses, stepping from a holding potential of −40 mV to levels ranging from +40 to −60 mV, with 20 mV increments in control conditions, after development of a stable effect of histamine (H), histamine plus DPH, DPH alone and after the drug washout. (b) Mean ± SEM current density versus voltage (I-V) curves, corresponding to all mentioned conditions. The * and ‡ indicate a statistically significant difference by multiple comparison vs. control group at each membrane potential (One Way ANOVA and Holm-Sidak post hoc analysis, n = 4–8, p < 0.05).

We first examined the effect of DPH on whole-cell currents mediated by Hv1 channels in Jurkat T cells, depicted in Fig. 1. Extracellular perfusion of 0.1 mM DPH significantly inhibited the Hv1 current (Fig. 1a), achieving its maximal effect between 3 and 5 minutes. Moreover, 0.1 mM histamine affected neither the Hv1 current nor its inhibition by DPH. The effect of DPH was partially reversible (around 80%) in all tested cells after 15 min. (One Way ANOVA and Holm-Sidak post hoc analysis, n = 4–8, p < 0.05).

**Figure 2.**
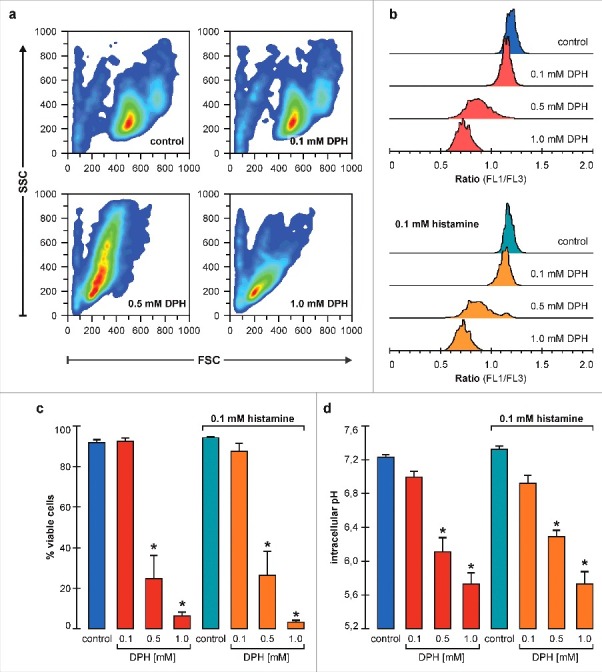
DPH effects on Jurkat T cells intracellular pH and viability. (a) Representative dot-plots of cells after 24 hours treatment with the indicated conditions, showing size (FSC) and complexity (SSC). Equivalent conditions in the presence of 0.1 mM histamine exhibited the same pattern (data not shown). (b) Representative histograms of the FL1/FL3 Ratio (proportional to pH_i_) for the same conditions shown in (a), where a shift toward left implies an intracellular acidification. (c) Mean values for % of viable cells in each condition. Asterisks refer to a statistically significant difference by Holm-Sidak post hoc analysis vs. control. (d) Mean values for intracellular pH in each condition. Asterisks refer to a statistically significant difference by Holm-Sidak post hoc analysis vs. control n = 5–6, p<0.05.

We next evaluated the effect of long-term DPH mediated Hv1 inhibition on pHi. Jurkat T cells were incubated with 0.1, 0.5 and 1.0 mM DPH for 24 hours alone or in presence of 0.1 mM histamine (experimental conditions used by Jangi et. al.^3^ to demonstrate DPH pro-apoptotic effects). Then pHi, cell size and complexity were simultaneously analyzed by flow cytometry and the results are presented in Fig. 2.

As it can be seen, in 0.5 and 1 mM DPH conditions a clear change in cell size and complexity (a typical phenomenon of the apoptotic process) occurred in parallel with pHi acidification (Fig. 2a and b, respectively). Considering this shift, we evaluated the percentage of viable cells observing a concentration dependent decrease unaffected by the presence of 0.1 mM of histamine (Two Way ANOVA and Holm-Sidak post hoc analysis, n = 5–6, p<0.05, see mean values Fig. 2c).

Simultaneously, fluorescence emission of the pH sensitive ratiometric probe BCECF was measured in the same cells, where a decrease in the values of FL1/FL3 ratio means intracellular acidification (Fig. 2b). Moreover, using the calibration curve performed in each experiment, FL1/FL3 ratio values were used to calculate the mean pHi value for each population (Fig 2d). The latter figure shows that DPH, in parallel with viability impairment, induced a significant dose dependent acidification that was also unaffected by 0.1 mM histamine (Two Way ANOVA and Holm-Sidak post hoc analysis, n = 5–6, p<0.05). Neither the viability nor the pHi showed differences in control conditions (0 mM DPH) with or without 0.1 mM histamine (Student's t-test, p<0.05). These results are coherent with our patch-clamp recordings, where the presence of 100 µM of histamine did not modify Hv1 currents or the inhibition exerted by DPH.

## Discussion

We previously demonstrated that Zn^2+^ and ClGBI, two Hv1 channel blockers, induced apoptosis of Jurkat T cells following a profound intracellular acidification.^22^ Now, in this work, we describe that DPH also inhibited proton currents in these leukemic cells, resulting in a drop on pHi in the same conditions where apoptosis was described previously.^3^ Our initial hypothesis has thus been reinforced by the results obtained with another compound that inhibits Hv1 channels, and the observed effect on pHi and apoptosis were similar to the ones observed with Zn^2+^ and ClGBI.

Our results and those reported by Jangi *et al.* showed the same concentration-dependent behavior in a range of concentration where the fractional occupancy of the H1-receptor is constant (remaining above the 99,9%, calculated assuming a pK_i_ = 7.9, IUPHAR DB) suggesting that DPH effects were not a consequence of H1 histamine receptor blockade.

In addition, the facts that both human Jurkat T cells and murine BV12 microglial cells^23^ exhibit the same percentage of proton current DPH inhibition with the same concentrations, as well as the short period of time action needed (3–5 min.) let us speculate that a direct interaction Hv1-DPH is involved in DPH pro-apoptotic effects.

Altogether, Hv1 inhibition is reinforced as a pro-apoptotic stimulus and a mechanism plausible to clarify the unexplained anti-tumorigenic properties of certain drugs.

## Methods

### Cell culture

Jurkat T cells were grown in DMEM High Glucose (25 mM) medium supplemented with 10% (vol/vol) heat-inactivated fetal bovine serum (Internegocios), in 5% CO2/95% humidified air at 37°C at an average density of 10^6^ cells per ml.

### Patch clamp experiments

The cells were observed with a mechanically stabilized, inverted microscope (Telaval 3, Carl Zeiss, Jena) equipped with a 40x objective lens. The standard tight-seal whole-cell configuration of the patch-clamp technique was used to record macroscopic whole-cell currents.^24^ Pipettes were drawn from capillary glass (PG52165–4, WPI, Boca Raton, Fla., USA) on a 2-stage vertical micropipette puller (PP-83, Narishige, Tokyo, Japan) and pipette resistances were 2–4 MΩ measured in extracellular solution. Ionic currents were measured with an appropriate amplifier (Axopatch 200A, Axon Instruments, Foster City, Calif., USA). Whole-cell currents were filtered at 2 kHz, digitized (Digidata 1440, Molecular Devices, LLC. Orleans Drive Sunnyvale CA, USA) at a sample frequency of 10 kHz and stored on a computer hard disk for later analysis. Total cell membrane capacitance was estimated by integrating the capacitive current transient elicited by the application of 10-mV hyperpolarizing step pulse from a holding potential of −60 mV. The estimated membrane capacitance of Jurkat T cells was 8.5 ± 2.8 pF (n = 18). All the experiments where performed using an agar-salt bridge.

Application of test solutions was performed through a multibarreled pipette positioned close to the cell investigated. After each experiment on a single cell, the experimental chamber was replaced by another one containing a new sample of cells. All experiments were performed at room temperature (∼22°C).

The extracellular saline solution (ESS) used for recording H^+^ currents contained (in mM): 100 4-(2-Hydroxyethyl) piperazine-1-ethanesulfonic acid (HEPES), 2 MgCl_2_.6H2O, 90 N-Methyl-D-glucamine (NMDG), 1 ethyleneglycol-bis(b-aminoethylether)-N,N,N,´N-tetraacetic acid (EGTA) and pH adjusted to 7.8 with HCl. The composition of the intracellular pipette solution (IPS) containing (in mM): 100 MES, 2 MgCl2.6H2O, 90 NMDG, 1 EGTA and pH adjusted to 6.3 with HCl.

For Hv1 blockade experiments, DPH solutions were made adding appropriate amounts of 100 mM aqueous stock solution to the ESS the same day of the experiment.

### Flow cytometry pHi determinations

Cells were incubated in 96-well plates (200 μl/well) at a starting concentration of 0.5 × 10^6^ cells/ml and cultured in the conditions abovementioned (see “Cell culture” section). Cells were exposed for 24 h to DPH 0.1, 0.5 and 1.0 mM before flow cytometry measurements. As DPH and histamine have been dissolved in aqueous media, control condition is simply medium addition in the same volume as stimulus.

The protocol described in Current Protocols in Cytometry (1997)^25^ was used for pH measurement with BCECF using Pseudo Null Calibration (also depicted by P. Frank et al^26^ and D. A. Eisner et al.,^27^ among others). Briefly, after incubation cells were centrifuged 5 min at 500 rpm and loaded with 2 μg/ml BCECF-AM 15 min at 37°C, centrifuged and resuspended in 10% FBS-HEPES solution. Prior measurement every batch of cells were exposed to the corresponding treatment condition at the same concentration of incubation to prevent eventual pHi recovery. Pseudo Null Calibration curve was performed according to Chow et al^25^ in each experiment (points pH = 8.0/7.7/7.4/7.1/6.8). The fluorescence of BCECF was monitored by a FACSCalibur flow cytometer (Becton-Dickinson) for an amount of 20.000 cells per tube. Data were acquired with CellQuest Pro 5.2.1 program and further analyzed with Flowing Software v2.5.1 (byPerttuTerho, Turku Center for Biotechnology, Finland) software. A two order polynomial fitting between Ratio of FL1/FL3 channels vs. calibration pH values was performed for each experiment, the output equation was later used to calculate pHi in each condition.

### Viability determinations

Simultaneous to FL1 and FL3, FSC and SSC intensity was acquired from the same batch of cells of pHi determinations; these parameters reflect cell size and granularity, respectively. Typically, during apoptosis progression cells reduce its volume and increase its intracellular complexity, so the percentage viable cells has been estimated as those that remains in the FSC/SSC gate were control cells commonly reside.

### Statistics

The results are expressed as mean ± standard error of the mean (SEM). Paired or unpaired Student's t tests were used to compare 2 groups. ANOVA (One Way or Two Way) test was used to compare 3 or more groups. In all cases, a P value lower than 0.05 was considered for establishing statistically significant differences.

### Reagents

Two′,7′-Bis(2-carboxyethyl)-5(6)-carboxyfluoresceinacetoxymethyl ester (BCECF-AM) was obtained from Invitrogene (Invitrogen Corporation, USA). All other reagents are from Sigma-Aldrich (St. Louis, MO) unless otherwise indicated. DMEM medium and FBS were purchased from local suppliers.

## Abbreviations

BCECF-AM: 2′,7′-Bis(2-carboxyethyl)-5(6)-carboxyfluoresceinacetoxymethyl ester
CA: Carbonic anhydrase
cAMP: cyclic adenosine 3′,5′-monophosphate
cGMP: cyclic guanine 3′,5′-monophosphate
ClGBI: 2-(6-chloro-1H-benzimidazol-2-yl) guanidine
DMEM: Dulbecco's Modified Eagle's Medium.
DPH: Diphenhydramine
FL1 and FL3: Fluorescence detectors 1 (530 nm) and 3 (661 nm), respectively
FSC: forward scatter detector
H: histamine
Hv1: Voltage gated proton channel
IUPHAR DB: International Union of Basic and Clinical Pharmacology Database
MCT: Monocarboxylate transporters
NBC: Sodium bicarbonate co-transporter
NHE: Sodium-proton exchanger
NMDA: N-Methyl-D-aspartate
pHi: Intracellular pH
SSC: side scatter detector
V-ATPase: Vacuolar type H^+^ ATPase
